# Summed Tissue Resistance of Periodontal Ligaments and Alveolar Bone in Orthodontic Distal Retraction of Maxillary Canines: Mathematical Simulation of Clinical Data and Interpretation of Results

**DOI:** 10.3390/dj13020055

**Published:** 2025-01-27

**Authors:** Olimpia Bunta, Vlad Muresan, Dana Festila, Mihaela Baciut

**Affiliations:** 1 Orthodontics Department, Faculty of Dental Medicine, Iuliu Hațieganu University of Medicine and Pharmacy, 400012 Cluj-Napoca, Romania; 2 Automation Department, Faculty of Automation and Computer Science, Technical University of Cluj-Napoca, 400114 Cluj-Napoca, Romania; vlad.muresan@aut.utcluj.ro; 3 Maxillofacial Surgery and Implantology Department, Faculty of Dental Medicine, Iuliu Hațieganu University of Medicine and Pharmacy, 400012 Cluj-Napoca, Romania

**Keywords:** orthodontic tooth movement, canine distal retraction, mathematical model, elastomeric chain, periodontal ligaments, alveolar bone, orthodontic biomechanics

## Abstract

**Background**: The mechanical properties of either alveolar bone or periodontal ligaments under orthodontic loading, as well as orthodontic tooth movement, have been studied in recent years using computational approaches. In previous studies, we developed a theoretical mathematical approach that uses a weighting coefficient of the summed resistance of periodontal structures, namely the bone and periodontal ligaments, in relation to apex movement, the center of rotation, orthodontic force loading, and time in order to quantify the biological response to orthodontic biomechanics. **Methods**: We analyzed the distal retraction of three maxillary canines and integrated the clinical data obtained in the previously developed mathematical programs. **Results**: The values of the (σ) weighting coefficient of the tissue resistance were interpreted in the context of the clinical data obtained: the smaller the value of (σ), the higher the actual tissue resistance, with a greater difference between the crown and root movement; also, the higher the value of (σ), the lower the actual tissue resistance, with a small difference between the crown and apex movement. **Conclusions**: The clinical interpretation of the results allows us to set a premise for the refinement of the mathematical programs so that we can use them in assessing the orthodontic biomechanics of larger patient groups over longer periods of time and create premises of treatment protocol simplification and adjustment.

## 1. Introduction

The behavior of periodontium structures, namely periodontal ligaments (PDLs) and alveolar bone, has been intensely studied in the context of orthodontic tooth movement [1,2,3,4,5,6,7,8,9,10,11,12]. As orthodontic treatments often involve premolar extractions and the distal retraction of canines, understanding the mechanisms of these phenomena creates the premises for more adapted clinical protocols. There are multiple factors involved in orthodontic tooth movement: age, sex, tooth morphology, bone anatomy, PDL anatomy, and the mechanical aspects of the orthodontic treatment, such as the material of the orthodontic appliance, the bracket prescription, the bracket slot dimensions, the bracket placement on the buccal surface of the tooth crown, the arch wire dimensions, friction, and the amount and line of action of the orthodontic force. Given all these aspects, the rate of response to treatment for each individual is different, and an exact treatment duration cannot be formulated before the beginning or during the treatment.

As there are multiple factors involved in the orthodontic dynamics of tooth movement, research can assess these factors mostly on an individual basis by analyzing their behavior in certain predetermined conditions. For instance, given the complexity of periodontal structures, anatomically and physiologically, the vast majority of studies in the literature, in vitro or in vivo, have approached the behavior of either PDL or bone separately, as it is very difficult to analyze them both at the same time.

Over the last years, more and more computational studies, the majority of them using the Finite Element Method/Analysis (FEM/FEA) [13,14,15,16,17,18,19], have tried to explain and elucidate the phenomena of orthodontic tooth movement, with the purpose of developing more efficient and shorter treatments [20,21,22,23,24,25,26,27,28].

The FEM represents a numerical technique used with the purpose of analyzing and simulating physical phenomena, such as fluid behavior, the growth of cells in a biological context, and mechanical stress [29,30]. From a medical perspective, the use of the FEM is helpful in research of complex biomechanical systems, which are difficult to evaluate through in vivo and in vitro approaches [29]. By incorporating structure, geometry, and material properties into an FEA, the models developed can simulate the mechanical behavior of bones by means of different loading scenarios [31,32]. Following a similar approach, studies regarding PDL behavior under orthodontic force loading have been used in order to assess the mechanisms of these structures in orthodontics, especially in the context of limited in vivo studies.

Even though it is considered a very useful tool in biomedical research, the FEM has its limitations, and its accurateness is directly related to the acknowledgement of these limits. These limits are related to the acknowledgement and use of adequate failure criteria, anatomical boundaries of simulation conditions, the anatomical response to mechanical loading, and the accuracy of 3D anatomical models. On the other hand, being a purely in vitro research tool, an FEA does not have the ability to reproduce complex clinical orthodontic movements [33].

The computational approach has offered a more accurate overview of the particular behavior of periodontal structures under orthodontic loading and applied force, but further studies are still needed in order to include in the analysis all the parameters implicated in these movements.

Given this broad context of orthodontic tooth movement and the various factors involved in these phenomena, in our previous studies, we developed a mathematical model using an approximating solution for solving differential equations with partial derivates, which reproduces the orthodontic tooth movement behavior during treatment [34]. The theoretical mathematical model proposed has the advantage of incorporating more of the implicated factors in these biomedical movements [35], as this approach represents, in fact, the mathematical integration of physical processes and phenomena, which determines the exact solution to the problem in the context of the precise knowledge of the initial data involved. Due to the fact that orthodontic processes are distributed parameter processes, they can be modeled using partial differential equations.

**Figure 1:**
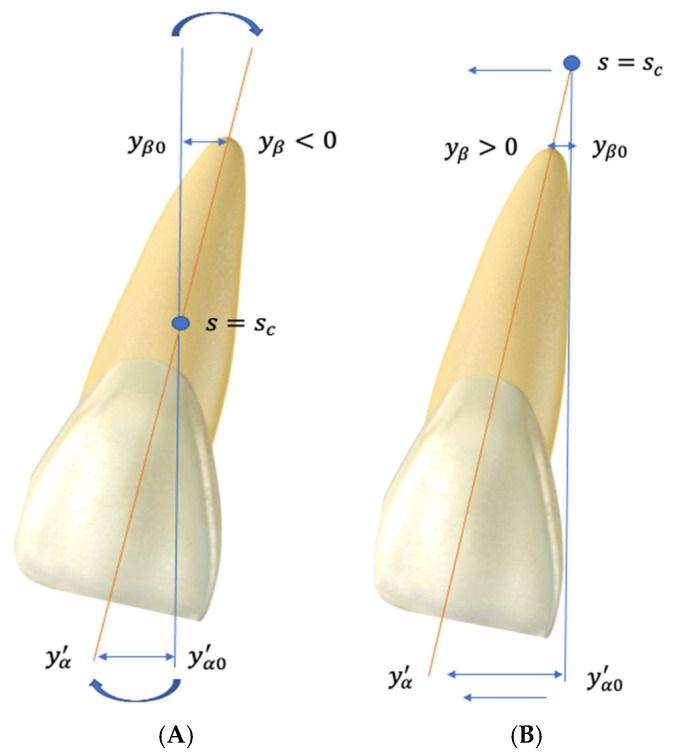
Possible orthodontic movements: (**A**) rotation; (**B**) roto-translation.

After generating the mathematical model, our initial research addressed the orthodontic tooth movement of the central maxillary incisor due to its simplified anatomy and intuitive overlapping with an approximating elliptic parabolic. As generally acknowledged in orthodontics, a pure translation movement of a tooth is impossible to achieve clinically due to the application of force at the level of the crown, which is far from the center of resistance of the tooth. In order to have a clear image of the possible outcomes, we defined the two tooth movement variants as rotation, with crown movement in one direction and apex movement in the opposite direction, and roto-translation, with crown and apex movement in the same direction, but in different degrees, as shown in Figure 1.

Tooth morphology was integrated in the mathematical model using the following notations: $y_{\alpha }^{\prime }$—incisal edge movement, $y_{\beta }$—apex movement, ${\tilde{s}}^{\prime }$—distance from the bracket slot to the incisal edge of tooth, $\tilde{s}$—distance from the bracket slot to the gingival margin, $s_{f}^{\prime }$—total length of the tooth (from incisal edge to apex), and $s_{f}$—length from the gingival margin to the apex.

The initial orthodontic force $\left(u_{0}\right)$ was introduced as an initial parameter in the model, as well as the remanent orthodontic force ${(u}_{f})$ of the considered elastomeric chains used to determine the orthodontic tooth movement.

By integrating time (t) and space $\left(s\right)$ constants into the mathematical model, we were able to determine the $\left(K_{y}\right)$ proportionality coefficient of the analyzed movement, making so the connection between the $u_{0}\left(t\right)$ input signal determined by the initial orthodontic force and the $y_{00}^{\prime }\left(t,s\right)$ output signal, which represents the actual tooth movement. In other words, by integrating all of these parameters in the mathematical model, we were able to simulate the tooth movement in predetermined initial and final conditions [34,35].

Based on the integration of the approximating solution of the proposed model, we then determined the center of rotation ${(s}_{c})$ for the two possible tooth movements—rotation and roto-translation—in relation to the ${(y}_{\beta })$ position of the apex. The next step in our research was to determine the (σ) weighting coefficient of the tissue resistance, which from a clinical point of view sums the PDL and bone biomechanical characteristics. The weighting coefficient proposed represents the total value of the plastic moment of the tissue reacting force, with unimportant variation in relation to time.

After simulation of the rotation movement of the central maxillary incisor, we interpreted the results of the (σ) weighting coefficient. From the theoretical interpretation of the rotation movement, we concluded that the lower the value of the (σ) weighting coefficient, the higher the value of the tissue resistance with placement of the rotation center outside the tooth volume, and the higher the value of the (σ) weighting coefficient, the lower the value of the tissue resistance, with placement of the rotation center inside the tooth volume [34,35].

By introducing the (σ) coefficient as the weighting coefficient of the environmental resistance of the periodontium structures in relation to the apex movement, center of rotation, the orthodontic force loading, and the time variable, in our previous studies, we determined an accurate mathematical model of the orthodontic tooth movement [34,35]. The (σ) weighting coefficient of the plastic response of the tooth supporting tissues represents a novel and unique approach in orthodontic tooth movement research, simplifying this very complicated and elaborate phenomena. Our overall study purpose was, from the beginning, to set the premises of a mathematical approach that includes the main factors implicated in this biomechanical therapeutic act in order to generate the actual dynamics of the process and create refinement premises, if needed.

As this was theoretical research that used theoretical predetermined initial and final parameters in order to assess the orthodontic tooth movement, clinical data were needed in order to determine the accuracy and adaptability of the mathematical model. Due to the frequency of premolar extractions in orthodontic treatments, we decided to clinically analyze the distal retraction of the maxillary canine with the purpose of integrating the clinical data into the proposed mathematical model and verify the accuracy of the simulations and that of the interpretation of the (σ) weighting coefficient.

## 2. Materials and Methods

### 2.1. Ethical Approval and Considerations

The protocol of this study was reviewed and approved by the Ethics Committee of the “Iuliu Hatieganu” University of Medicine and Pharmacy, under number 587/10.12.2019. The participation of the patients in this study was completely voluntary, with the possibility of retraction of consent for participation at any given time during the study. Patients gave their informed consent for participation in this clinical data collection study, for using their data in studies related to orthodontic tooth movement dynamics, and also for publishing the results of the studies generated by the use of their data. Blinding was applied just to the outcome assessors, the names of the patients being concealed, and replaced with teeth numbering. As all patients were treated by one doctor; blinding of the main researcher and of the patients was not possible due to the nature of the therapeutic act.

### 2.2. Case Selection

For this study, we collected clinical data from 2 patients: one male, aged 19, and one female, aged 24.

The inclusion criteria for the case selection involved the following: non-growing patients; patients in a course of orthodontic treatment with fixed appliances; patients in the retraction stage of the upper canine; appliance prescription, Roth 022; patients that required orthodontic first upper premolars; patients with intra-alveolar temporary anchorage devices—TADs—positioned at the level of the interradicular space of the second upper premolar and the first upper permanent molar or between the first and the second upper permanent molars; patients with no general pathologies and medication; patients without previous orthodontic treatments; and patients that attended all their appointments, according to protocol, during the study.

The exclusion criteria for this study were as follows: growing patients; patients with removable orthodontic appliances/aligners; patients in the alignment and leveling stages of orthodontic treatment; appliance prescription, MBT 018 or MBT 022; patients that did not require orthodontic extractions or required orthodontic extractions of other teeth than the first upper premolars; patients without intra-alveolar TADs in the upper arch; and patients that did not attend their appointments, according to protocol, during this study.

### 2.3. Orthodontic Treatment Protocol

From the two patients, we analyzed the distal retraction of three maxillary canines: two belonging to the male patient, teeth C1 and C2, and one belonging to the female patient, tooth C3. The distal retraction phase of the treatment was consecutive to orthodontic first premolar extractions, in both patients, for all 3 maxillary canines analyzed. All 3 premolar extractions were performed before bonding of the fixed orthodontic appliance.

Both patients were undergoing active orthodontic treatment with fixed appliances, a Roth 022 prescription [Low Profile, American Orthodontics, AO, Sheboygan, WI, USA]. The examination of the movement of the analyzed teeth was made after the alignment and leveling stage, on 0.017 × 0.025-inch stainless steel wires, using memory elastomeric chains [American Orthodontics, AO, Sheboygan, WI, USA] as force vectors. The arch wire material and dimensions were chosen in accordance with the accepted clinical protocols for canine distal retraction on 022 prescription fixed orthodontic appliances. The memory elastomeric chains were stretched between the canine and the first molar brackets. Both patients had TADs in the maxillary arch, inserted in the upper vestibule between the roots of the first premolar and the first permanent molar or between the first and second permanent molars, in order to increase orthodontic anchorage.

### 2.4. Force Measurement

The measuring of the force was made using an NK-20 Analog Force Gauge (HUIOU, Shenzhen, China) at 4 different time intervals over the period of 2 activation cycles, each of 28 days. The first measurement was made on day 1, considered FI1, and then day 14, considered Fint1, of the first activation cycle of the 28 days.

After the first activation cycle, we measured the movement of the 3 canines, and a new memory elastomeric chain was used, stretched between the same brackets—the canine and first molar. The force generated by the new memory elastomeric chain was measured again on day 1 of the second activation cycle, considered FI2, and on day 14 of the same activation cycle, considered Fint2.

### 2.5. Canine Movement Measurements

The position of the maxillary canines was determined in relation to the TADs inserted in the upper posterior areas of the arch. The TADs were considered fixed points, as there were no mobility issues at this level. The position of the canines was assessed after the two activation cycles: CA1, first activation cycle, and CA2, second activation cycle.

The movement of the crown was determined at the level of the tip of the canine cuspid$\;{(y}_{\alpha }^{\prime }$) in relation to the fixed point—the TAD. The apex movement ($y_{\beta }$) was determined by analyzing the long axis of the canines and bone and root morphology emphasizing the clinical and intraoral root movement of the analyzed teeth.

Intraorally, we made measurements on the distance between the canine bracket slot and the tip of the canine cuspid (${\tilde{s}}^{\prime }$) between the canine bracket slot and the gingival margin ($\tilde{s}$) and the canine bracket slot and the tooth apex ($s_{f}$). The determination of the total length of the tooth, crown, and root was also determined on digital panoramic radiographs. The digital panoramic X-rays (Planmeca, Helsinki, Finland) were created on a scale of 1:1, using the following parameters: 73.0 [kVp], 10.0 [mA], and 1.23 [dGyCm^2^].

### 2.6. Data Analysis

The results obtained after these clinical measurements on patients undergoing active orthodontic treatment were transposed into the mathematical model previously developed [34,35].

Based on the relation

(1) $$y_{\beta }=\frac{s_{c}-(s_{f}+\tilde{s})}{s_{c}+{\tilde{s}}^{\prime }}\cdot y_{\alpha }^{\prime }$$

from which the mathematical result is

(2) $$y_{\beta }\cdot s_{c}+y_{\beta }\cdot {\tilde{s}}^{\prime }=s_{c}\cdot y_{\alpha }^{\prime }-(s_{f}+\tilde{s})\cdot y_{\alpha }^{\prime }$$

(3) $$s_{c}\cdot \left(y_{\alpha }^{\prime }-y_{\beta }\right)=y_{\beta }\cdot {\tilde{s}}^{\prime }+\left(s_{f}+\tilde{s}\right)\cdot y_{\alpha }^{\prime }$$

so as

(4) $$s_{c}=\frac{y_{\beta }\cdot {\tilde{s}}^{\prime }+(s_{f}+\tilde{s})\cdot y_{\alpha }^{\prime }}{y_{\alpha }^{\prime }-y_{\beta }}$$

and based on

(5) $$\sigma =\sigma (t,s)=\frac{u_{0}(t)\cdot s_{c}}{\delta_{0}\cdot s_{f}+\frac{\delta_{1}}{2}\cdot \left(s_{f}^{2}+2\cdot s_{f}\cdot \tilde{s}\right)+\frac{\delta_{2}}{3}\cdot (s_{f}^{3}+3\cdot s_{f}^{2}\cdot \tilde{s}+3\cdot s_{f}\cdot {\tilde{s}}^{2})}$$

we generated the rotation center$\;{(s}_{c})$ of the 3 maxillary canines and also the weighting coefficient of the tissue resistance for the analyzed teeth.

## 3. Results

The dental parameters relevant to this study (determined through intraoral measurements), the forces generated intraorally by the memory elastomeric chains, and the dental movements (assessed at the level of the canine tip of cusp and at the level of the canine apex, over the two activation cycles, as compared to their initial positions) are presented in Table 1.

The median values of the rotation center evolution and the instant values of the (σ) weighting coefficient for all three analyzed teeth during the two activation cycles are presented in Table 2.

## 4. Discussion

In this study, we analyzed intraorally the orthodontic movement of three maxillary canines in two patients undergoing active treatment: one male, teeth C1 and C2, and one female, tooth C3. The present study was conducted as part of doctoral research [35].

### 4.1. Force Analysis

The most frequent means of distal retraction in orthodontics are elastomeric chains and nickel–titanium closed coil springs. Even if some consider nickel–titanium closed coil springs to be more efficient due to their mechanical properties, continuous force delivery, and low degradation rates, studies have shown that on average, these are only 0.2–0.5 mm per month faster at closing spaces than plastic elastomeric chains [36,37]. On the other hand, discomfort is greater with nickel–titanium closed coil springs, and the oral hygiene in much more difficult to maintain [37]. Based on this information and since we have previously studied in vitro the behavior of elastomeric chains, our choice of force input for this study was a memory elastomeric chain.

The orthodontic forces generated by the memory elastomeric chains that were used in the active treatment of the two abovementioned patients are presented in Table 1 and are compared to the degradation rates obtained in vitro in our previous studies [38]. Our in vitro comparative study of the plastic and memory orthodontic chains of the producer American Orthodontics showed a less substantial and slower degradation rate of the memory elastomeric chain, with our results agreeing with other studies in the literature [38]. The results of the in vitro comparative study determined our decision to use memory elastomeric chains in the clinical protocol of the present study. The literature acknowledges the impact of external and internal factors on the evolution of elastomeric materials in the oral cavity; therefore, a comparative analysis between the in vitro and in vivo behavior of these materials was made.

**Figure 2:**
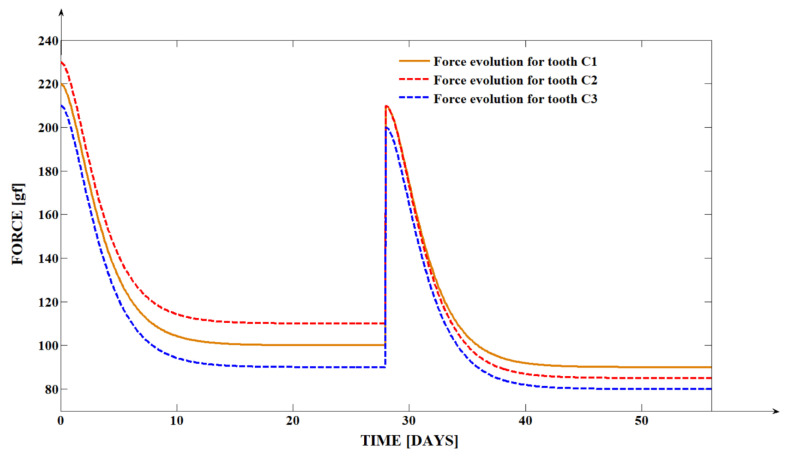
Intraoral evolution of the force generated by the memory elastomeric chains used in this study.

The differences between the initial generated forces, comparing the in vitro (mean value of 347 g force) [38] and in vivo studies (mean value of 220g force), are due to the necessity of lower forces in vivo. The use of high forces during orthodontic treatments might lead to high sensibility and even pain and also to the possible negative effects of such high forces on the periodontal structures, tooth structure, and vitality. Comparing the degradation rates of the forces generated by memory elastomeric chains in vitro and in vivo, during the first 14 days, the differences are small. The memory elastomeric chain presents much more stable intraoral behavior, being influenced a small amount by external factors (mouthwash, beverages ingested, and food). This is a very important clinical fact due to the constantly descendant evolution of the force, as can be observed in Figure 2. This evolution favors a more linear and unitary movement of the two extremities, the tooth apex and the tooth tip of the cuspid, so a more roto-translation movement rather than a rotational one.

### 4.2. Dental Movement Analysis

Distal canine retraction is recommended after the alignment and leveling phases of arches. As found in the specialty literature, the range of orthodontic canine distal retraction at the level of the cusp of the tooth is between 0.62 and 1.15 mm/month [39,40]. Drawing a parallel to the study performed by Zheng and Yang in 2021, with a mean canine distal retraction in their control group of 0.85 ± 0.23 mm at the end of a 4-week period [39], the median results of our distal canine retraction over the same time interval was 0.83 mm.

Studies in the literature present multiple options for the main arch wire selection, especially when using orthodontic fixed appliances with 022-inch slots. The arch wire dimensions vary between 0.016 × 0.022 inch, 0.017 × 0.025 inch, 0.018 × 0.025 inch, and 0.019 × 0.025 inch, but there is no variation between the material choice, with stainless steel being the accepted option in terms of rigidity and proper control of tooth movement [39,40,41]. The main examiner, who treated the patients included in this study, chose 0.017 × 0.025-inch stainless steel arch wires for this stage of treatment.

**Figure 3:**
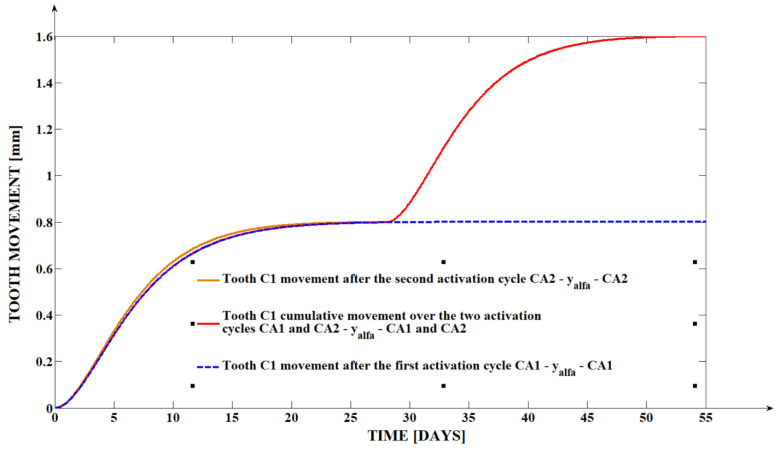
The $y_{\alpha }^{\prime }$ evolution over the period of the two activation cycles for tooth C1.

**Figure 4:**
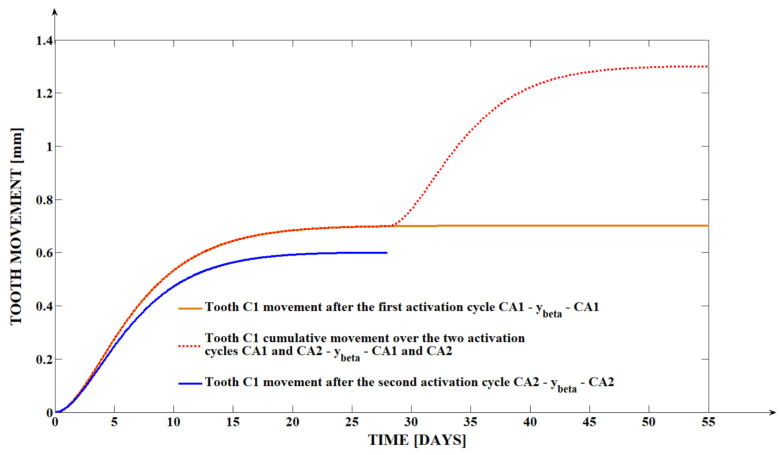
The $y_{\beta }$ evolution over the period of the two activation cycles for tooth C1.

The dental movements obtained from the clinical measurements at the level of the tip of the canine cuspid ($y_{\alpha }^{\prime }$) and of the canine apex$\;{(y}_{\beta })$ for the three analyzed teeth under the form of a roto-translation can be seen in Table 1. For a better visual interpretation of the clinical data, we created in the mathematical programs graphical representations of the distal retraction evolution of tooth C1, both at the level of the tooth tip of the cusp ($y_{\alpha }^{\prime }$) and the apex$\;\left(y_{\beta }\right)$, as shown in Figure 3 and Figure 4.

The evolution of the apex movement is obviously being left behind the crown movement, highlighting the roto-translational nature of the analyzed movement. The fact that ${(y}_{\beta })$ presents a smaller value for all three cases at the end of the first activation cycle is the direct result of the fact that the orthodontic force is applied at the level of the tooth crown, at a distance from the resistance center of the tooth. The fact that the apex movement$\;{(y}_{\beta })$ presents an even smaller value at the end of the second activation cycle compared to its values at the end of the first activation cycle can be explained through the same phenomena, especially because the second activation cycle begins at a moment in which the tooth has already been displaced inside the alveolar bone, with ${(y}_{\beta })\;$ being already behind ${(y}_{\alpha }^{\prime })$ at the end of CA1. Because the force is applied at the level of the tooth crown and the crown has no obstacles in its path, $y_{\alpha }^{\prime }>y_{\beta }$.

### 4.3. Rotation Center and (σ) Weighting Coefficient of the Tissue Resistance Analysis

Based on relations (4) and (5), we generated in the mathematical programs designed specifically for orthodontic tooth movement dynamics [34] the evolution of the ($s_{c}$) rotation center and of the (σ) weighting coefficient of the tissue resistance for the case of the three canines analyzed, which presented a roto-translation type of movement.

It is important to mention the fact that the tooth movement type is influenced by the (σ) weighting coefficient of the tissue resistance and the applied force, and that it directly influences the position of the ($s_{c}$) rotation center.

Also, (σ) represents a weighting coefficient of the tissue resistance, not being the actual resistance of the periodontium, and is expressed in grams force/square millimeter [grf/mm^2^].

In Table 2, we can observe the mean values of the ${(s}_{c})$ rotation center and the instant values of the (σ) weighting coefficient of the tissue resistance for all three maxillary canines analyzed for the duration of the two activation cycles of 56 days in total. The instant values of the (σ) weighting coefficient of the tissue resistance were generated for precise moments in time.

**Figure 5:**
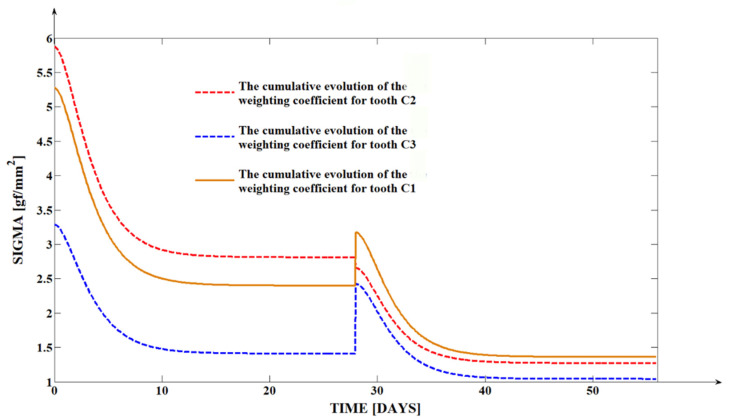
The (σ) weighting coefficient evolution for the 3 analyzed teeth.

The graphically comparative representation based on the results of the simulation, Figure 5, highlights the discontinuity of the curves, which is due to the generation of the instant values of the (σ) weighting coefficient of the tissue resistance generated by the calculus. As mentioned before, the instant values of the (σ) weighting coefficient were generated for precise moments in time but also in relation to the initial input signals—initial force. When addressing graphically the whole time interval analyzed, the curves present an evolution proportional to the evolution of the force generated by the memory elastomeric chains.

As observed in Figure 5, the curves related to the second activation cycle present a less pronounced evolution than the curves related to the first activation cycle and also a tendency towards stabilization. In the event of analyzing ulterior activation cycles, with intraoral measurements, we can consider the stabilization of the curves. The clinical interpretation of the biomechanical phenomena of the analyzed teeth movements, with a pronounced evolution of the curves for CA1 and a remissive decreasing evolution of the curves for CA2, stands for the reaction of the supporting tissues of teeth—the periodontal structures immediately after orthodontic force loading with memory elastomeric chains.

The (σ) weighting coefficient of the tissue resistance presents values inside the [1.6634–3.2254] interval for all three teeth analyzed at the end of CA1.

The value 3.2254 gf/mm^2^ of the (σ) weighting coefficient, for the C2 maxillary canine, can be interpreted as a reduced resistance of the periodontal structures. The value 2.7884 gf/mm^2^ has a similar interpretation, as both values are over σ=2 gf/mm^2^. The σ=2 gf/mm^2^ calculation threshold was generated in the previous theoretical simulations of the program when introducing the (σ) coefficient in the transcendent equation of equilibrium between the plastic resistance of the environment in relation to the elastic moment generated by the elastomeric chain [34].

These values can be interpreted in the context of higher dental parameters for the C1 and C2 maxillary canines rather than for the C3 maxillary canine: both C1 and C2 belong to the male patient. For the case of the C3 maxillary canine, the value σ=1.6634 gf/mm^2^ represents an increased resistance of the periodontal structures, even though the dental parameters for this tooth are slightly reduced; the tooth belongs to a female patient.

The evolution of the (σ) weighting coefficient of the tissue resistance for the second activation cycle, CA2, shows quite reduced values inside the interval [1.1129–1.6072] for all three of the maxillary canines analyzed in this study. The (σ) variation for CA2 can be correlated with the dental movement values for the same time interval, when ${(y}_{\beta })$ remains even more behind ($y_{\alpha }^{\prime }$) than in CA1. The stabilization of the curves in CA2 can be interpreted as being the reaction of the periodontal structures to consecutive force delivery by the memory elastomeric chain, which presents a remissive decreasing evolution, automatically having the same effect over a longer period.

Given the fact that the bone resorption process on the side of the traction happens in the first days after force delivery, the bone apposition process requires a longer time interval, and because the tooth crown has no obstacles on its displacement, the ${(y}_{\beta })$ evolution involved by the (σ) weighting coefficient is justified.

Our previous studies computationally analyzed the theoretical behavior of a rotation type movement for the case of a maxillary central incisor. The present study computationally analyzed the in vivo behavior of a roto-translation type movement for the case of maxillary canines.

For better understanding of the results of the two types of movements, Table 3 presents an interpretation of the rotation center and that of the weighting coefficient based on the results generated in the mathematical programs.

**Figure 6:**
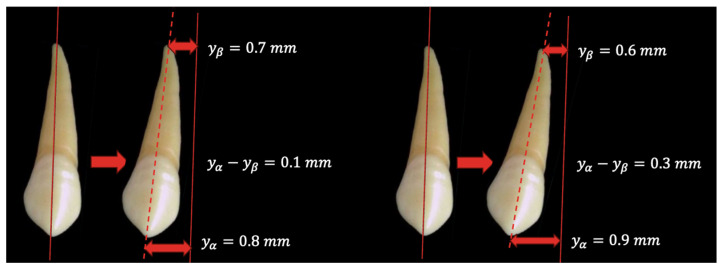
The different degrees of movement between the tooth crown and apex.

For the initial theoretical simulations, we considered a tooth with a total length of 24 mm and an initial force of 100gr. For the present study, the tooth parameters and the initial force are those presented in Table 1. Because the (σ) weighting coefficient of the tissue resistance is generated based on relation (5), in relation to the initial force, and the dental parameters $\left(s_{f}\right)$ and $\left(\tilde{s}\right)$ are determined in relation to $\left({\tilde{s}}^{\prime }\right)$, it is obvious that any modifications in these parameters are responsible for the tooth movement inside the periodontal tissues. The difference in tooth movement between the initial theoretical simulation and the one performed using clinical data is that the apex moves in the same direction as the crown but in a smaller amount, thus highlighting the roto-translation character of the movement, as shown in Figure 6.

Based on our results so far, we can state the fact that there is a small variation in the interpretation of the apex movement in relation to the (σ) weighting coefficient of the tissue resistance due to different dental parameters (maxillary central incisor vs. maxillary canine), different initial values of the applied forces, and different types of movement (rotation vs. roto-translation).

For the rotation movement, we can observe an inversely proportional relationship between the values of the (σ) weighting coefficient of the tissue resistance and the actual tissue resistance. This means that the smaller the value of (σ), the higher the actual tissue resistance; therefore, it is responsible for the negative position of the tooth apex and a rotation type of movement. And also, the higher the value of (σ), the lower the actual tissue resistance; therefore, it is responsible for the smaller negative degree of the tooth apex and a tendency towards a roto-rotation type of movement [34].

For the roto-translation movement, we can observe the same inversely proportional relationship between the (σ) weighting coefficient of the tissue resistance and the actual tissue resistance. This means that the smaller the value of (σ), the higher the actual tissue resistance; therefore, it is responsible for the tendency of the root apex to remain behind the crown movement but still have positive values, so it is also a roto-translation type of movement. And also, the higher the value of (σ), the lower the actual tissue resistance; therefore, it is responsible for the positive position of the tooth apex with a small difference between the crown and apex movements.

Based on the theoretical result of our previous studies, but mostly the results of the present study, which used clinical data from patients undergoing orthodontic treatments, we can state that even in different loading conditions, i.e., different initial force values, the programs developed for the orthodontic tooth movement dynamics are remarkably accurate mathematically, are highly adaptative and clinically reproductible, and create premises for multiple and various future studies.

### 4.4. Limitations of the Study and Future Research

The limitations of the present study are the reduced number of patients without statistical assessment, the short duration of the study, and the clinical determination of the apex movement.

For a more obvious interpretation of the (σ) weighting coefficient of the tissue resistance values, refinements should be performed within the mathematical programs so as to obtain a directly proportional relation between the coefficient and the actual tissue resistance.

Further studies on larger groups over a longer time interval and with radiological (CBCT) measurements of the apical movement could open the possibility of a classification of the summed periodontal resistance of PDLs and alveolar bone. It also creates premises for developing a digital library of data that can help clinicians refine their treatment protocols in relation to the (σ) weighting coefficient of the tissue resistance.

## 5. Conclusions

For the present study, even though the sample of analyzed teeth was small, our purpose was to address the overall intraoral behavior as compared to the initial theoretical findings and create premises of mathematical model refinement for use on larger patient samples in the future.

The introduction and interpretation of the (σ) weighting coefficient of the tissue resistance values represent a novel approach of the biomechanical processes that orthodontic treatments generate. The interpretation and transposing in the mathematical model of the clinical measurements from patients undergoing active orthodontic treatment has confirmed the theoretical digital biomechanics of the orthodontic tooth movement dynamics previously developed.

The (σ) weighting coefficient of the tissue resistance represents a tool for a better understanding and simplification of orthodontic biomechanics, with multiple and various future research possibilities.

## Figures and Tables

**Table 1:** Dental parameters, evolution of force generated by the memory elastomeric chain, and dental movement.

Maxillary Canine	Patient	$s^{\prime }f$ [mm]	${\tilde{s}}^{\prime }$ [mm]	$\tilde{s}$ [mm]	$s_{f}$ [mm]	FI1 [gr/f]	Fint1 [gr/f]	$y_{\alpha }^{\prime }$ [mm] CA1	$y_{\beta }$ [mm] CA1	FI2 [gr/f]	Fint2 [gr/f]	$y_{\alpha }^{\prime }$ [mm] CA2	$y_{\beta }$ [mm] CA2
C1	Male	31	5	4.5	21.5	220	100	0.8	0.7	210	90	0.8	0.6
C2	Male	30	5	4.5	20.5	230	110	0.8	0.7	210	85	0.9	0.6
C3	Female	29.5	4.5	4.5	20.5	210	90	0.9	0.7	200	80	0.9	0.6

Legend: $s^{\prime }f$—total length of tooth, measured from the tip of the canine cuspid to the apex; ${\tilde{s}}^{\prime }$—distance between the bracket slot and the tip of the canine cuspid; $\tilde{s\;}$—distance between the bracket slot and the gingival margin; $s_{f}$—length of the tooth from the gingival margin to the apex; FI1—measurement of force on day 1 of the first activation cycle; Fint1—measurement of force on day 14 of the first activation cycle; FI2—measurement of force on day 1 of the second activation cycle; Fint2—measurement of force on day 14 of the second activation cycle; $y_{\alpha }^{\prime }$—movement of the tip of the canine cuspid; $y_{\beta }$—movement of the canine apex.

**Table 2:** Median values of the $\left(s_{c}\right)$ evolution and instant values of the (σ) evolution for the 3 analyzed teeth.

Maxillary Canine	$s_{c}$ [mm] CA1	σ [gf/mm^2^] CA1	$s_{c}$ [mm] CA2	σ [gf/mm^2^] CA2
C1	243	2.7884	119	1.6072
C2	235	3.2254	85	1.1780
C3	128.25	1.6634	84	1.1129

Legend: $s_{c}$—rotation center; σ—weighting coefficient of the tissue resistance; CA1—first activation cycle; CA2—second activation cycle.

**Table 3:** Comparative interpretation of the results.

Movement Type	$s_{c}$ [mm]	σ [grf/mm^2^]
Rotation	Inside the tooth morphology	High values (>2 grf/mm^2^)—reduced tissue resistance Low values (<2 grf/mm^2^)—increased tissue resistance
Roto-translation	Outside the tooth morphology	High values (>2 grf/mm^2^)—reduced tissue resistance Low values (<2 grf/mm^2^)—increased tissue resistance

Legend: $s_{c}$—rotation center; σ—weighting coefficient of the tissue resistance.

## Data Availability

The data presented in this study are available upon request from the corresponding author.

## References

[1] Li Y., Zhan Q., Bao M., Yi J., Li Y. (2021). Biomechanical and biological responses pf periodontium in orthodontic tooth movement: Up-date in a new decade. Int. J. Oral Sci..

[2] Perillo L., d’Apuzzo F., Illario M., Laino L., Di Spigna G., Lepore M., Camerlongo C. (2020). Monitoring biomechanical and structural changes in human periodontal ligaments during orthodontic treatment by means of micro-Raman Spectroscopy. Sensors.

[3] Toyama N., Ono T., Ono T., Nakashima T. (2023). The interleukin 6 signal regulates orthodontic tooth movement and pain. Biochem Biophys. Res. Commun..

[4] D’Apuzzo F., Nucci L., Delfino I., Potaccio M., Minervini G., Isola G., Serino I., Camerlino C., Lepore M. (2021). Application of vibrational spectroscopies in the quantitative analysis of gingival crevicular fluid and periodontal ligament during orthodontic tooth movement. J. Clin. Med..

[5] Wang K., Xu C., Xie X., Jing Y., Chen P.J., Yadav S., Wang Z., Taylor R.W., Wang J., Feng J.Q. (2022). Axin2+ PDL cells directly contribute to new alveolar bone formation in response to orthodontic tension force. J. Dent. Res..

[6] Pustulka K., Trzcionka A., Dziedzic A., Skaba D., Tanasiewicz M. (2021). The radiological assessment of root features and periodontal structures in endodontically treated teeth subjected to forces generated by fixed orthodontic devices. A prospective, clinical cohort study. J. Clin. Med..

[7] Motyl S., Manfredini D., Oruba Z., Bugajska J., Sztefko K., Stos W., Osiewicz M., Loster B.W., Lobbezoo F. (2021). Evaluation of interleukin-1 beta and the ration of interleukine-1 beta to interleukine-1 receptor antagonist in gingival crevicular fluid during orthodontic canine retraction. Dent. Med. Probl..

[8] Roth C., Craveiro R.B., Niedarau C., Malyaran H., Neuss S., Jankowski J., Michael W. (2022). Mechanical compression by simulating orthodontic treatment tooth movement in an in vitro model modulates phosphorylation of AKT and MAPKs via TLR4 in human periodontal ligament cells. Int. J. Mol. Sci..

[9] Kukreja B.J., Bhat K.G., Kukreja P., Kumber V.M., Balakrishnan R., Govila V. (2021). Isolation and immunohistochemical characterization of periodontal ligament stem cells: A preliminary study. J. Indian Soc. Periodontol..

[10] Yan L., Liao L., Su X. (2022). Role of mechano-sensitive non-coding RNAs in bone remodeling of orthodontic tooth movement: Recent advances. Prog. Orthod..

[11] Chen L., Yu H., Li Z., Wang Y., Jin S., Yu M., Zhu L., Ding C., Wu X., Wu T. (2024). Force-induced Caspase-1-dependant pyroptosis regulates orthodontic tooth movement. Int. J. Oral Sci..

[12] Rizck M., Niederau C., Florea A., Kiessling F., Morgenroth A., Mottaghy F.M., Schneider R.K., Wolf M., Craveiro R.B. (2023). Periodontal ligament and alveolar bone remodeling during long orthodontic tooth movement analyzed by a novel user-independent 3D-methodology. Sci. Rep..

[13] Moga R.A., Olteanu C.D., Buru S.M., Botez M.D., Delean A.G. (2023). Cortical and trabecular bone stress assessment during periodontal breakdown—A comparative Finite Element Analysis of multiple failure criteria. Medicina.

[14] Prados-Privado M., Martinez-Martinez C., Gehrke S.A., Prados-Frutos J.C. (2020). Influence of bone definition and Finite Element Parameters in bone and dental implant stress: A literature review. Biology.

[15] Moga R.A., Olteanu C.D., Botez M.D., Buru S.M. (2023). Assessment of the orthodontic external resorbtion resorption in periodontal breakdown-a Finite Element Analysis (part I). Healthcare.

[16] Moga R.A., Olteanu C.D., Daniel B.M., Buru S.M. (2023). Finite Element Analysis of totth tooth—A comparative analysis of multiple failure criteria. Int. J. Environ. Res. Public Health.

[17] Bandela V., Basanu R., Nagarajappa A.K., Basha S., Kanaparthi S., Ganji K.K., Patil S., Gudipanedi R.K., Mohammed G.S., Alam M.K. (2021). Evaluation of stress distribution and force in external hexagonal implant: A 3D finite element analysis. Int. J. Environ. Res. Public Health.

[18] Ortun-Terrazas J., Cegonino J., Santana-Penin U., Santana-Mora U., Perez del Palomar A. (2019). A porous fibrous hyperelastic damage model for human periodontal ligament: Application of a microcomputerized tomography finite element model. Int. J. Number. Method Biomed. Eng..

[19] Petrescu S.M., Tuculina M.J., Popa D.L., Duta A., Salan A.I., Georgescu R.V., Diaconu O.A., Turcu A.A., Mocanu H., Nicola A.G. (2022). Modeling and simulating an orthodontic system using virtual methods. Diagnostics.

[20] Suzuki M., Sueishi K., Katada H., Togo S. (2019). Finite element analysis of stress in maxillary dentition during en masse retraction with implant anchorage. Bull. Tokyo Dent. Coll..

[21] Yamaguchi M., Fukasawa S. (2021). Is inflammation a friend or foe for orthodontic treatment? Inflammation on orthodontically induced inflammatory root resorbtion and accelerating tooth movement. Int. J. Mol. Sci..

[22] Abbing A., Koretsi V., Eliades T., Papageorgiou S.N. (2020). Duration of orthodontic treatment with fixed appliances in adolescents and adults: A systematic review with meta-analysis. Prog. Orthod..

[23] Kiyamehr Z., Razeghinejad M.H., Rahbar M., Oskouei S.G., Vafaei A. (2022). Factors affecting the duration of fixed orthodontic treatment in patients treated in a university department between 2016 and 2020. Maedica.

[24] Jung M.H. (2021). Factors influencing treatment efficiency. A prospective cohort study. Angle Orthod..

[25] Wazwaz F., Seehra J., Carpenter G.H., Ireland A.J., Papageorgiou S.N., Cobourne M.T. (2022). Duration of tooth alignment with fixed appliances: A systematic review and meta-analysis. Am. J. Orthod. Dentofac. Orthop..

[26] Niemi P., Kortelainen M., Harjunmaa U., Waltimo-Siren J. (2023). Costs and duration of orthodontic-surgical treatment with mandibular advancement surgery. Eur. J. Orthod..

[27] Ke Y., Zhu Y., Zhu M. (2019). A comparison of treatment effectiveness between clear aligner and fixed appliance therapies. BMC Oral Health.

[28] Papageorgiou S.N., Koletsi D., Iliadi A., Peltomaki T., Eliades T. (2020). Treatment outcome with orthodontic aligners and fixed appliances: A systematic review with meta-analysis. Eur. J. Orthod..

[29] Falcinelli C., Valente F., Vasta M., Traini T. (2023). Finite element analysis in implant dentristry dentistry: State of the art and future directions. Dent. Mater..

[30] Stein E. (2014). History of finite element method—Mathematics meets mechanics—Part 1: Engineering developments. The History of Theoretical Material and Computational Mechanisms—Mathematics Meets Mechanics and Engineering.

[31] Varga P., Willie B.M., Stephan C.S., Kozloff K.M., Zysset P.K. (2020). Finite element analysis of bone strength in osteogenesis imperfecta. Bone.

[32] Currey J. (2009). Measurement of the mechanical properties of bone: A recent history. Clin. Orthop. Relat. Res..

[33] Moga R.A., Buru S.M., Olteanu C.D. (2022). Assessment of the best FEA failure criteria (part I): Investigation of the biomechanical behaviour behavior of PDL in intact and reduced periodontium. Int. J. Environ. Res. Public Health.

[34] Bunta O., Festila D., Muresan V., Colosi T., Stan O.P., Unguresan M.L., Baciut M. (2023). Mathematical modeling and digital simulation of teeth dynamics for the approximation of orthodontic treatment duration. Appl. Sci..

[35] Nemes O.M. (2024). Studies Regarding the Improvement of the Orthodontic and Orthognatic Tooth Movement Biodinamics. Ph.D. Thesis.

[36] Badran S.A., Al-Zaben J.M., Al-Taie L.M., Tbaishi H., Al-Omiri M.K. (2022). Comparing patient-centered outcomes and efficiency of space closure between nickel-titanium closed-coil springs and elastomeric power chains during orthodontic treatment: A two-center, randomized clinical trial. Angle Orthod..

[37] Mohammed H., Rizk M.Z., Waffaie K., Almuzian M. (2018). Effectiveness of nickel-titanium springs vs elastomeric chains in orthodontic space closure: A systematic review and meta-analysis. Orthod. Craniofac. Res..

[38] Bunta O., Muresan V., Pacurar M., Baciut M., Nenovici D., Varlam-Molnar C., Tarmure V. (2019). Mechanical strength variety of orthodontic polymeric chains: In vitro assessment and mathematical model. Mater. Plast..

[39] Zheng J., Yang K. (2021). Clinical research: Low-level laser therapy in accelerating orthodontic tooth movement. BMC Oral Health.

[40] Almaasarani S.G., Rahej N. (2023). A comparison of maxillary canine retraction into healed and recent extraction sites using cone beam computed tomography: A randomized clinical trial. Angle Orthod..

[41] Alfawazan A.A., Manas A., Verghese Y., Kochhar A.S., AlMogbel A.M., Patil S. (2022). A CBCT assessment of bone density changes after accelerated orthodontic retraction of canine by microosteoperforations. J. Orthod Sci..

